# Technical emptiability of dairy product packaging and its environmental implications in Austria

**DOI:** 10.7717/peerj.7578

**Published:** 2019-09-10

**Authors:** Bernhard Wohner, Nicole Schwarzinger, Ulla Gürlich, Victoria Heinrich, Manfred Tacker

**Affiliations:** Section Packaging and Resource Management, FH Campus Wien, Vienna, Austria

**Keywords:** Food residues, Food waste, Food loss, Emptiability, Sustainability, Circular economy, Milk, Product environmental footprint, Emptying, Streamlined LCA

## Abstract

**Background:**

Food waste is a major ecological concern around the globe. While the main function of packaging is to contain and protect food, it may also lead to food waste if residues remain in a package after emptying. Such residues could be attributed to wasteful behavior of consumers, but also to properties of packaging (e.g., geometry, surface tension) and food (e.g., surface tension, viscosity).

**Methods:**

In this study, the technical emptiability (ability of packaging to be emptied entirely) of 36 dairy products is analyzed. Firstly, the amount of food residues in packaging after emptying at room and refrigerator temperature was weighed and set in relation to the original filling quantity. Secondly, streamlined life cycle assessments (LCAs) based on the Product Environmental Footprint guidance with a functional unit of “one kg of consumed dairy product at room or refrigerator temperature in the home of the consumer” are conducted. Finally, technical emptiability was included in the streamlined LCA and attributed to the primary packaging in order to evaluate its environmental impact.

**Results:**

Technical emptiability for both temperatures combined was found to be between 0.25% (±0.11) and 5.79% (±0.43) for the analyzed dairy products. While there were differences in emptiability results of the same product and different temperatures, no significant trend (*p* = 0.94) between emptiability and temperature could be observed. Liquid yogurt, cream, and buttermilk in beverage cartons and plastic bottles yielded the highest amounts, while milk in beverage cartons and glass bottles yielded the lowest amounts regarding food residues. Looking at global warming potential, poor technical emptiability of cream in a beverage carton leads to even higher environmental impacts than the production and waste management of its packaging.

**Discussion:**

The streamlined LCA results show that food residues can contribute substantially to the footprint of packaging and can have similar or even higher environmental impacts than packaging production and waste management. Yet, emptiability is remarkably under-researched to this day. Future studies should further develop the methods presented in this paper, while LCA analysts should include technical emptiability when assessing the sustainability of packaging, particularly for those containing resource-intensive goods.

## Introduction

Worldwide, 1.3 billion metric tons or approximately one-third of the food produced is lost or wasted every year (Gustavsson, Cederberg & Sonesson, 2011). Food losses and waste (FLW) account to the emission of 3.3 billion tons of CO_2_ equivalents and, when compared to countries, is ranked as the third top emitter after USA and China (Food and Agriculture Organization of the United Nations (FAO), 2013).

In theory, optimized packaging can reduce both food and packaging waste across the supply chain (Food and Agriculture Organization of the United Nations (FAO), 2014; Verghese et al., 2015), for example, by providing mechanical protection (Oki & Sasaki, 2000) or by using modified atmosphere packaging and thus prolonging the shelf life of its contents (Kirtil & Oztop, 2016). In low and middle-income countries, missing or inappropriate packaging is stated as one of the major contributors to FLW (Food and Agriculture Organization of the United Nations (FAO), 2014). In contrast, in Europe or North America more food is wasted at the consumption stage (Gustavsson, Cederberg & Sonesson, 2011). Here, packaging can be directly responsible for FLW due to various reasons (Wohner et al., 2019), for example:

- Inappropriate packaging size, that is, too large packages
- Packaging that is difficult to open
- Packaging that is not reclosable
- Packaging that is difficult to empty

However, how exactly and to what extent packaging functions influence FLW is still largely unexplored (Wikström et al., 2019). In total, packaging may be responsible for up to 25% of FLW in households (Williams et al., 2012). According to this study, packaging that is “difficult to empty” is identified as a major driver of FLW. Further, “emptiability” (ability of emptying a package completely) is stated as particularly important for reducing FLW of yogurt (Wikström et al., 2019).

Several consumer protection agencies and companies are already concerned with emptiability (Markert, 2016; Austrian Association for Consumer Information (VKI), 2017; LiquiGlide Inc, 2018). Still, existing scientific literature on this subject is scarce. For instance, Meurer et al. (2017) detail their approach of emptying UHT milk, while in other literature the emptiability of yogurt (Williams et al., 2012) and minced meat in trays or tubes (Wikström, Williams & Venkatesh, 2016) are stated, yet without the description of a reproducible methodology for quantification. Despite the aforementioned studies, emptiability can be considered under-researched, even though it may lead to relevant environmental impacts. More specifically, by food residues interfering with the recycling of packaging (Packaging SA, 2017; Maris et al., 2018), as well as by the unnecessary resource consumption and emissions related to the production of food (Food and Agriculture Organization of the United Nations (FAO), 2013). As a rule, food production has considerably higher environmental impacts than its packaging (Silvenius et al., 2011; Licciardello, 2017). Therefore, a resource-intensive packaging can actually have a lower environmental impact than a resource-extensive one if it leads to less FLW (Denkstatt, 2014). Yet, many life cycle assessment (LCA) studies of packaging exclude packaging-related FLW, while the awareness of its importance is fortunately increasing (Molina-Besch, Wikström & Williams, 2018).

To fill the identified literature gap, this paper addresses the question on how to quantify emptiability, or more precisely, technical emptiability. In this context, technical emptiability is considered as the sole product of the respective food packaging combination while excluding any wasteful behavior a consumer might practice. Furthermore, the study discusses if the attribution of food residues leads to the conclusion that technical emptiability testing should be carried out and included in the LCA of packaging. For this purpose, streamlined LCAs of all products are performed, that is, refraining from the collection of primary data.

The present research is restricted to dairy products since these are particularly resource-intensive (Clune, Crossin & Verghese, 2017) and are consumed in large quantities in Austria (Statistik Austria, 2018).

## Materials and Methods

### Testing of technical emptiability

A total of 36 dairy products were purchased from various brands in several Austrian supermarkets.

In addition to the packaging geometry, emptiability is mainly influenced by the surface tension of food and packaging, along with the viscosity of food (Schmidt, 2011). Moreover, viscosity changes with temperature (Gonçalves et al., 2017) and dairy products are usually consumed both directly after removal from the refrigerator, as well as on the go after they have gained room temperature. Therefore, tests were performed at room (22 ± 1 °C) and refrigerator temperature (7 ± 1 °C).

While the testing for milk was based on Meurer et al. (2017), due to the lack of scientific literature a new methodology for emptying dairy products other than milk had to be adapted or rather newly developed. A pre-test was carried out to observe how long the content actually flows and then drips out, similar to Meurer et al. (2017). After this preliminary test, a total emptying time of 2 min including a shake of the package was chosen, since after that no more dripping of milk occurred. While emptying, the “perfect consumer” was simulated, that is, emptying with meticulous precision, so that the derived emptiability could actually be attributed to the packaging and not to a potentially wasteful consumer behavior. As a result, the emptying of packaging was carried out until it became apparent that no more food could be removed from the packaging without damaging it.

The principal steps of testing were (i) weighing of the package (food and packaging), (ii) emptying the contents, (iii) weighing the emptied package, (iv) washing and air drying of the packaging for 48 h at room temperature (22 ± 1 °C) and (v) weighing of the cleaned packaging.

The mass of food residues in a package (FR_*i*_) was then calculated as:

$${\rm{FR}}_{i} = {\rm{Emptied\ package}}_{i} - {\rm{Cleaned\ packaging}}_{i}$$

Due to time and resource restraints resulting from the analysis of 36 products, a number of three tests per temperature and package was chosen. The emptiability index (EMPT) of the respective temperature was the arithmetic mean of all three emptiability tests per package and temperature, expressed as the ratio of FR_*i*_ to the mass of the food in a package (F_*i*_):

$${\rm{EMPT}}_{\rm{Temp}}(\%)={\sum\nolimits_{i = 1}^3 {\rm{FR}}_{i}\over \sum\nolimits_{i = 1}^3 {\rm{F}}_{i}}\times 100$$

Since it is not known at which temperature the dairy products are consumed in practice, the discussion focuses more on the mean of both temperatures. As a result from the formula, a lower emptiability index means a better emptiability of packaging. Subsequently, statistical reliability of the derived emptiability results were analyzed by power tests (Cohen, 1988) in order to calculate variability. First, a desired statistical power of 0.80 with a confidence interval of 95% was defined. This resulted in effect sizes (Cohen’s d) of 3.26 for three samples and of 1.44 for six samples. Finally, variability regarding emptiability indices was defined as these values multiplied with the respective standard deviation (Data S1).

#### Emptiability testing of different types of milk, buttermilk, and chocolate milk

For the emptying of milk (whole milk, low-fat milk, lactose-free skimmed milk), buttermilk and chocolate milk, the packaging was held upside down and kept in this position for 1 min. The packaging was then brought to the starting position, panned five times and held for 10 s. Finally, it was tilted again and held for 1 min upside down. For chocolate milk in a beverage carton, the emptying was carried with the provided straw by pressing the package.

#### Emptiability testing of café latte

For café latte, the cup was shaken five times and then opened, whereby the intended drinking lid was put on for emptying. Further emptying followed the same procedure as for milk.

#### Emptiability testing of cream and low-fat cream alternative

Cream and low-fat cream alternative in bottles were emptied similar to milk variations. The multilayer polymer pouch for low-fat cream alternative was cut open at the designated area and its contents were squeezed out.

#### Emptiability testing of liquid yogurt

In addition to the emptying method of milk, the packages of liquid yoghurt were shaken five times before being opened.

#### Emptiability testing of yogurt, sour milk, fresh, and curd cheese

For all yogurt, sour milk, and cheese products, both the packaging was spooned out and the lid scraped off with a spoon. For the emptying process always the same spoon was used, which was washed and dried between each measurement.

### Streamlined life cycle assessment

#### Goal and scope definition

The goal of the streamlined LCA is to understand the relative impact of including emptiability in LCA of primary packaging. In contrast to full LCAs, streamlined LCA omit the collection of primary data.

As the dairy products are sold, consumed and disposed of in Austria and generally contain Austrian dairy, the geographical area chosen is also Austria.

The methodology for carrying out LCA is based on the current guidance (version 6.3) for the product environmental footprint (PEF) (European Commission, 2017) and the product environmental footprint category rules (PEFCR) for dairy products (Bengoa, Dubois & Humbert, 2018) in particular. The calculations are performed with OpenLCA 1.7.4 and the Ecoinvent 3.5 database. All used datasets are listed in the Data S2.

#### Functional unit and reference flow

The functional unit chosen is “one kg of consumed dairy product at room or refrigerator temperature in the home of the consumer.” The reference flow is the amount of product needed to fulfil the functional unit. As an example, this results in a reference flow of 1,010 g for a product with a filling quantity of 1,000 g and an emptiability-related loss of 10 g.

#### System boundaries

**Figure 1:**
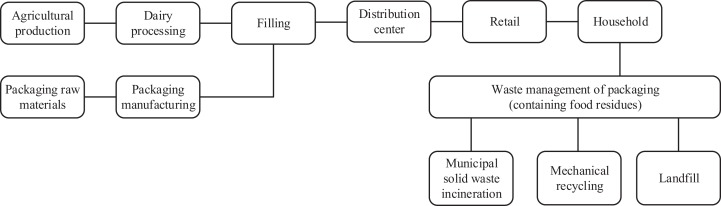
System boundaries of streamlined life cycle assessment.

The system boundaries of the streamlined LCA include the raw materials, manufacturing and transport of packaging with all its components, the agricultural production and processing of milk and other ingredients, as well as the disposal of the packaging including the food residues inside (Fig. 1).

The included transports are those of the packaging and the ingredients to the filling plant, as well as those of the product from the dairy plant to the distribution center, then to the supermarket and finally to the consumer. Transport distances are taken from the PEFCR and are listed in the Data S2.

Not included in the streamlined LCA are the final assembly of the packaging (e.g., application process of an aluminum lid to a plastic cup) and the use phase (e.g., energy consumption of the refrigerator), as well as FLW at other food supply chain stages. The refrigeration process in the household has not been considered since the packaging design does not affect the energy consumption of the refrigerator. Therefore, according to the PEF guidance, this is to be classified as a product-independent use stage process and shall thus be excluded from the system boundary.

#### Life cycle stages

Life cycle stages are calculated and listed separately (Data S3) for

- Primary packaging (PRP): raw materials, production, transport, and waste management
- Food production (F): production of dairy products
- Waste management of food residues (FW): waste management (incineration) of food residues in packaging after emptying
- Secondary/tertiary packaging (STP): raw materials, production, transport, and waste management
- Transport to home: transportation of packages from the supermarket to the home of the consumer

The attribution of food residues to the LCA of the respective primary packaging follows a similar approach detailed in Wikström, Williams & Venkatesh (2016). Subsequently, a newly derived result of each impact category for the respective primary packaging after including food residues (PRP_FR*i*_) is calculated. For this, the environmental impacts regarding the production and waste management of (i) STP, (ii) the production of food, (iii) the transport of the products to the home of the consumer (TH_*i*_) (iv) and that of the waste management of food residues (FW_*i*_) was attributed to the production and waste management of primary packaging (PRP_*i*_).

$${\rm{PRP}}_{{\rm{FR}}_{i}}= {\rm{PRP}}_{i} + {{\rm{PRP}}_{i} + {\rm{STP}}_{i} + {\rm{TH}}_{i} + F_{i}\over 1-{\rm{EMPT}}_{i}} \times {\rm{EMPT}} + {\rm{FW}}_{i}$$

For EMPT, the mean of all six emptiability tests was used, since the temperature at which the products are consumed was not known. Finally, PRP_FR_ was compared to the LCA results based purely on production and waste management of the primary packaging (PRP_*i*_).

#### Selection of impact categories

All 16 impact categories of the PEF (ILCD 2.0 2018 impact categories set) were calculated and listed for all investigated life cycle stages in Data S3. For the interpretation, however, only the most relevant impact categories were used. For this purpose, first the results of all impact categories are normalized and weighted (Data S3). Next, the absolute values of all but the toxicity categories are added to obtain the PEF single score. Toxicity categories were excluded since they are not yet robust enough (Bengoa, Dubois & Humbert, 2018; Sala, Cerutti & Pant, 2018). Finally, the most relevant impact categories were those which contribute at least 80% to the PEF single score. For this study, this results in a list of the following six categories, ranked by their contribution:

- Freshwater and terrestrial acidification (Accumulated Exceedance, in mol H^+^_eq_)
- Respiratory effects, inorganics (Impact on human health, in disease incidence)
- Climate change (Global Warming Potential over 100 years, in kg CO_2eq_)
- Terrestrial eutrophication (Accumulated Exceedance, in mol N^−^_eq_)
- Freshwater eutrophication (EUTREND model, in kg P_eq_)
- Resource use, fossils (Abiotic Resource Depletion, in MJ_eq_)

#### Life cycle inventory of packaging

First, each packaging was disassembled after the emptying process. Then, the packaging components were weighed and, finally, their material determined. Whenever the polymer type of plastic packaging was not recognizable by the label, its identification was carried out with Fourier-transform infrared spectroscopy.

**Figure 2:**
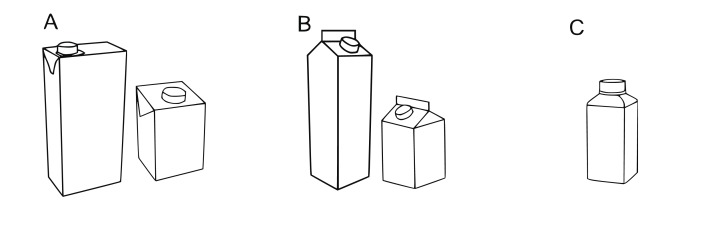
Emptied beverage cartons. (A) Cartons with flat tops. (B) Cartons with gable tops. (C) Cartons with bottle-shaped tops.

**Figure 3:**
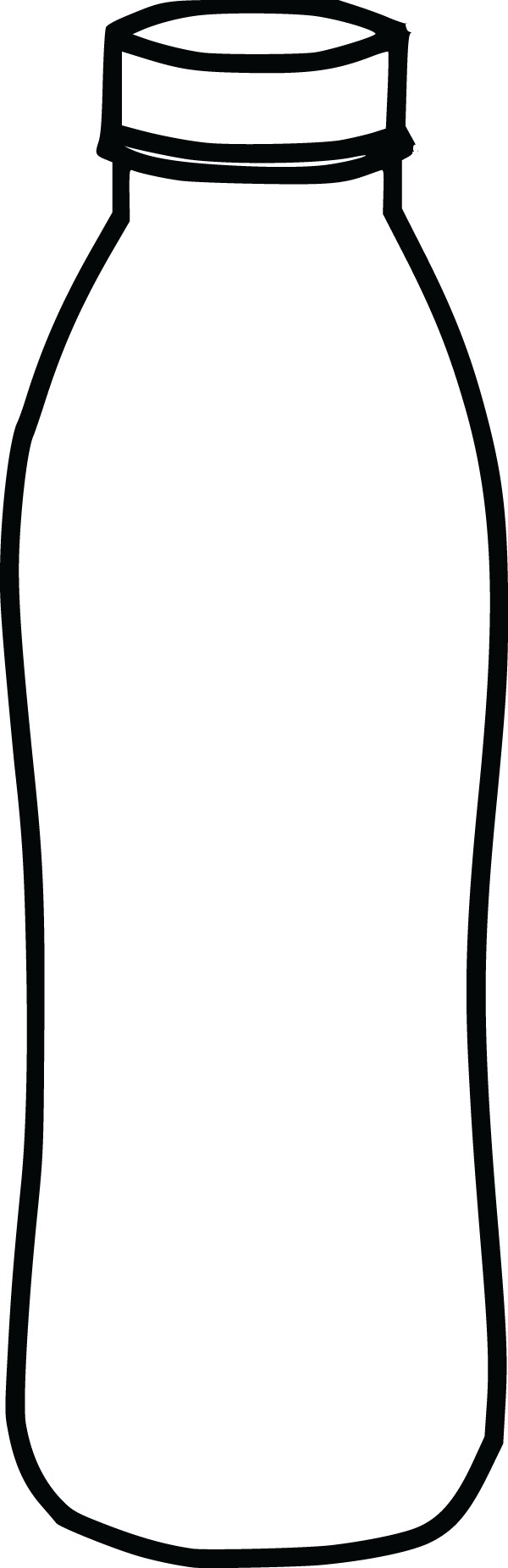
Emptied plastic bottles.

**Figure 4:**
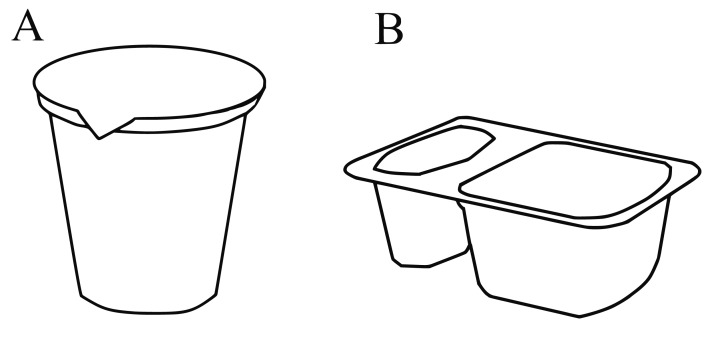
Emptied plastic cups. (A) Single chamber cups. (B) Twin-chamber cups.

**Figure 5:**
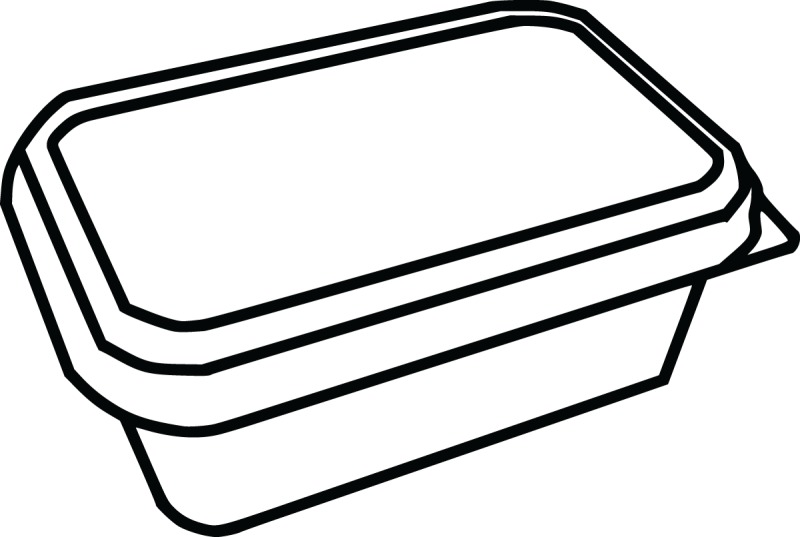
Emptied plastic tubs.

**Figure 6:**
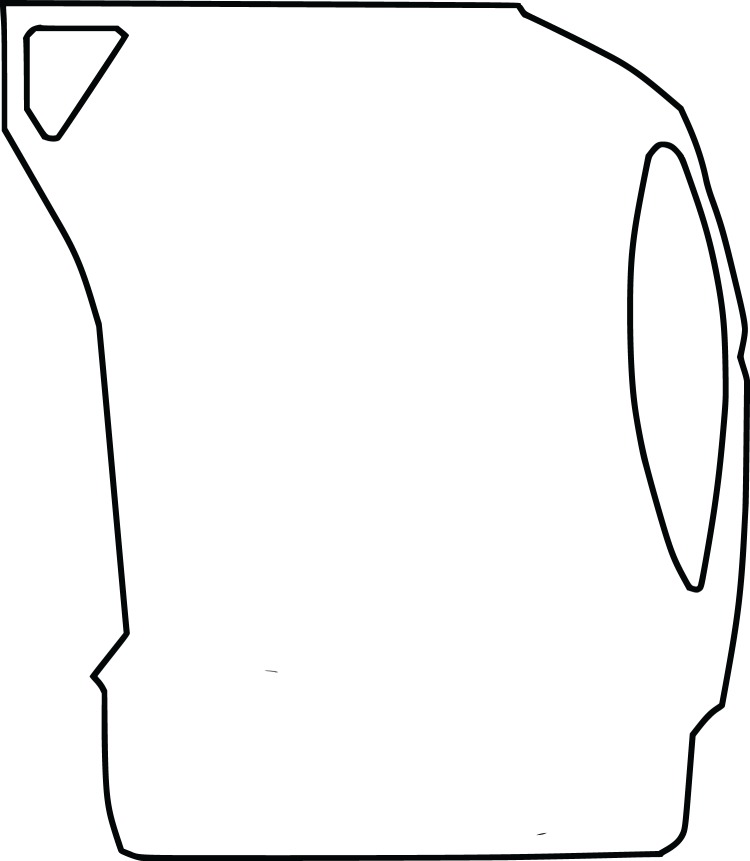
Emptied plastic pouches.

**Figure 7:**
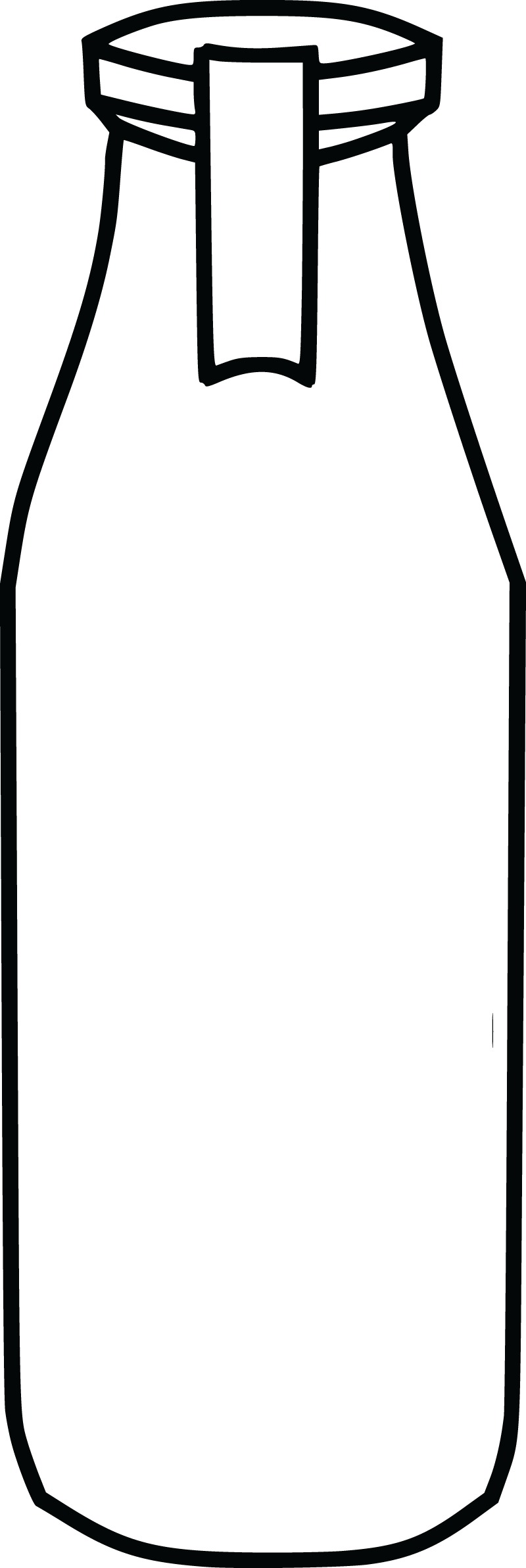
Emptied glass bottles.

Tested packaging consisted of:

- Aseptic and non-aseptic beverage cartons: with bottle-shaped, gable and flat tops (Fig. 2)
- Plastic bottles: high-density polyethylene (HDPE) and polyethylene terephthalate (PET) (Fig. 3)
- Plastic cups: polypropylene (PP), polystyrene (PS); single and twin-chamber cups (Fig. 4)
- Plastic tubs: PP and PS (Fig. 5)
- Pouch: multilayer polymer pouch (PP, PE, calcium carbonate, and ethylene vinyl alcohol (EVOH) (Fig. 6)
- Glass bottle: white packaging glass (Fig. 7)

Detailed packaging descriptions are listed in the Data S2. The composition of non-aseptic beverage cartons was assumed to be 80% cardboard and 20% low density polyethylene (LDPE), that of aseptic beverage cartons to be 75% cardboard, 21% LDPE and 4% aluminum (Fachverband Kartonverpackungen für flüssigkeits Nahrungsmittel eV (FKN), 2007). Zero recycled content was assumed for all materials, except for packaging glass, where 60% of recycled content was chosen (Austria Glas Recycling GmbH, 2018). The composition of the multilayer polymer pouch was taken from its environmental product declaration (Ecolean, 2018). Transport distances for glass and non-glass packaging to the filling plant were taken from Bengoa, Dubois & Humbert (2018) (Data S2), as well as default data for secondary and tertiary packaging (25.6 g corrugated board, 1.5 g LDPE film, and 6.0 g wooden pallet per kg dairy product).

#### Life cycle inventory of milk and dairy products

For the LCA of Austrian milk, methane (CH_4_) and nitrous oxide (N_2_O) emissions, as well as feed rations, were taken from the GLEAM tool provided by the Food and Agriculture Organization of the United Nations (FAO) (2018). The Austrian Air Pollution Inventory was used for information on ammonia (NH_3_) emissions from dairy cows (Anderl et al., 2018). A distance of 60 km was used for the transport of raw milk between dairy farms and processing units (Bengoa, Dubois & Humbert, 2018). Finally, a life cycle inventory for one kg of fat-protein corrected milk (FPCM) for Austria was modeled (Table S1). One kg of FPCM consists of 4.00% fat, 3.30% protein content and 4.85% lactose content (International Dairy Federation (IDF), 2015), which sums up to 12.15% milk solids.

The milk quantity required for each dairy product was then calculated according to the milk solids allocation (International Dairy Federation (IDF), 2015; Bengoa, Dubois & Humbert, 2018). Thus, the required amount of milk for the respective dairy product was calculated as the sum of its fat, protein and lactose content divided by 0.1215.

Recipes of processed products had to be estimated (Data S2) as the information on the package did not indicate exact quantities in most cases. Estimates were based on the imprinted list of ingredients and the nutrition labeling of the packed food. Information on energy and resource consumption for the processing of (i) milk, (ii) fermented products, and (iii) cheese was taken from Bengoa, Dubois & Humbert (2018). For café latte, the Ecoinvent dataset for green coffee beans was supplemented with data on grinding and roasting of coffee (Phrommarat, 2019).

#### End-of-life assumptions of analyzed products

The assumption that packaging is recycled can only be made if it is recyclable by design and if the packaging is actually collected, sorted and recycled in the respective country. Only then, a country-specific recycling rate for a type of packaging may be used. For these assessments, recyclability guidelines were used to determine the expected recycling rate of the specific products. As an illustration, while the recycling rate for PET bottles in Austria is 45% (Van Eygen, Laner & Fellner, 2018), the end-of-life assumption for white PET bottles is incineration, since such opaque bottles are not recyclable (Plastics Recyclers Europe, 2018). All examined PET bottles have opaque colors and are, therefore, not recycled in Austria. The HDPE bottle for cream (36% fat) has a full-body sleeve made of oriented PS and is therefore also not recyclable (Institute cyclos-HTP, 2017).

For plastic cups and tubs, it can be assumed that these are not recycled in Austria, as are small foils or pouches due to their size (Van Eygen, Laner & Fellner, 2018). Plastic cups that are wrapped with cardboard are seen as a multilayer packaging and hence, not recyclable, since the separation of the cardboard from the cup cannot be expected from the consumer (Tschachtli et al., 2018). Besides the packaging design, food residues of more than 1% by volume also affect the recycling of plastic bottles, cups, and foils (Packaging SA, 2017).

Thus, all analyzed plastic primary packaging is assumed to be incinerated. The only primary packaging that can be classified as both recyclable and recycled in practice are beverage cartons and glass bottles, with recycling rates of 30% (Getränkekarton Austria, 2019) and 86% (Altstoff Recycling Austria, 2018) respectively. Further, recycling rates of aluminum lids and closures are assumed to be 38% (Warrings & Fellner, 2018).

For the secondary and tertiary packaging, a recycling rate of 85% is assumed for cardboard (Altstoff Recycling Austria, 2018) and 39% for large LDPE films (Van Eygen, Laner & Fellner, 2018).

As there is a landfill ban on untreated waste in Austria (Austrian Federal Ministry of Agriculture, Forestry, Environment and Water Management (BMLFUW), 2008), it was assumed that no packaging, with the exception of glass and metal packaging, would be landfilled if it is not recycled.

#### Allocation rules

Allocation procedures follow the rules of the Circular Footprint Formula presented in the PEF guidance. Credits are awarded for the thermal and electrical energy gained from the incineration of the products, as well as for the recyclate resulting from recycling. Allocation and quality factors used in the PEF Circular Footprint Formula to calculate end-of-life burdens and credits are taken from the PEF default data (European Commission, 2019) and are also listed in the Data S2.

#### Robustness of the streamlined LCA results

For including technical emptiability in the streamlined LCA, the mean of all six EMPT tests was used. Subsequently, sensitivity analysis was carried out to evaluate potential implications resulting from emptiability indices that vary from the derived means. As a consequence, the calculations were repeated with the upper and lower limits of the determined variability of emptiability indices.

## Results

### Technical emptiability results

The determined emptiability results (7 and 22 °C combined) of all analyzed products amount to values between 0.25% (±0.11) and 5.79% (±0.43). Liquid yogurt in beverage cartons and PET bottles, as well as cream and buttermilk in beverage cartons have the poorest emptiability results (Table 1), while whole milk (Table 2) and crumbly curd cheese have better results in comparison. Emptiability indices (EMPT) for the various types of milk are found to be similar to the results of Meurer et al. (2017), with 0.31–0.45%. The derived variability of emptiability ranges between 0.03 (whole milk in a glass bottle) and 0.55% points (low-fat cream alternative in a polymer pouch), with a mean of 0.21. In percent, these values are between 2% (low-fat cream alternative in a PET bottle) and 70% (sour milk in a PP cup), with a mean of 20% (Data S1).

**Table 1:** Technical emptiability results for dairy products other than milk.

Dairy product	EMPT_22 °C_ (%)	EMPT_7 °C_ (%)	EMPT_22 °C, 7 °C_ (%)
Buttermilk | Beverage carton, bottle top, variant a	3.77 ± 0.77	4.18 ± 0.63	3.97 ± 0.42
Buttermilk | Beverage carton, bottle top, variant b	3.74 ± 0.44	4.13 ± 0.66	3.93 ± 0.38
Buttermilk | Beverage carton, gable top	3.32 ± 0.29	3.41 ± 0.17	3.36 ± 0.12
Cream, 23% fat | Beverage carton, flat top	4.10 ± 0.05	4.27 ± 1.00	4.18 ± 0.31
Cream, 36% fat | HDPE bottle	0.86 ± 0.13	0.80 ± 0.12	0.83 ± 0.07
Cream, 36% fat | PS cup	0.72 ± 0.12	0.60 ± 0.20	0.66 ± 0.11
Curd cheese, creamy | PS tub	0.79 ± 0.28	0.56 ± 0.20	0.67 ± 0.21
Curd cheese, crumbly | PS tub	0.27 ± 0.35	0.23 ± 0.17	0.25 ± 0.11
Fresh cheese, herbs | PP tub	0.53 ± 0.39	0.39 ± 0.19	0.46 ± 0.16
Fresh cheese, radish | PP tub, variant a	0.47 ± 0.32	0.48 ± 0.32	0.47 ± 0.13
Fresh cheese, radish | PP tub, variant b	0.49 ± 0.11	0.44 ± 0.20	0.46 ± 0.07
Fresh cheese, sweet pepper | PP tub	0.41 ± 0.10	0.40 ± 0.29	0.40 ± 0.09
Fresh cheese, sweetened | PS cup	0.44 ± 0.18	0.52 ± 0.20	0.48 ± 0.10
Liquid yogurt | Beverage carton, bottle top	4.40 ± 0.33	4.35 ± 0.62	4.38 ± 0.20
Liquid yogurt | Beverage carton, gable top	4.18 ± 1.13	4.18 ± 0.62	4.18 ± 0.36
Liquid yogurt, blueberries | PET bottle	5.95 ± 0.68	5.63 ± 1.06	5.79 ± 0.43
Liquid yogurt, strawberry | PET bottle	1.50 ± 0.26	1.36 ± 0.57	1.43 ± 0.21
Liquid yogurt, vanilla | PET bottle	1.66 ± 0.32	2.24 ± 0.25	1.95 ± 0.47
Low-fat cream alternative | PET bottle	3.86 ± 0.08	3.85 ± 0.28	3.85 ± 0.08
Low-fat cream alternative | Polymer pouch	1.44 ± 0.46	0.77 ± 0.32	1.10 ± 0.55
Sour milk | PP cup	0.35 ± 0.28	0.52 ± 0.03	0.43 ± 0.36
Sour milk | PS cup	0.40 ± 0.21	0.51 ± 0.26	0.45 ± 0.14
Yogurt, cereals | PS cup	0.67 ± 0.07	0.68 ± 0.16	0.68 ± 0.05
Yogurt, chocolate | PS cup	1.31 ± 0.93	1.04 ± 0.20	1.18 ± 0.34
Yogurt, fruits | Twin-PP cup	1.71 ± 0.89	1.72 ± 0.63	1.72 ± 0.30
Yogurt, vanilla | PS cup	0.94 ± 0.19	1.20 ± 0.53	1.07 ± 0.26

**Note:**

Results are given in percent, variability of results in percentage points.

**Table 2:** Technical emptiability results for milk and milk-based drinks.

Dairy product	EMPT_22 °C_ (%)	EMPT_7 °C_ (%)	EMPT_22 °C, 7 °C_ (%)
Cafe Latté | PET bottle	0.48 ± 0.08	0.57 ± 0.10	0.53 ± 0.08
Cafe Latté | PP cup	0.96 ± 0.81	1.54 ± 0.64	1.25 ± 0.54
Chocolate milk | Beverage carton, flat top	1.13 ± 0.19	1.40 ± 0.46	1.26 ± 0.25
Chocolate milk | PET bottle, variant a	0.86 ± 0.17	0.74 ± 0.36	0.80 ± 0.15
Chocolate milk | PET bottle, variant b	0.92 ± 0.11	1.06 ± 0.22	0.99 ± 0.13
L-free skimmed milk | Beverage carton, gable top	0.35 ± 0.08	0.35 ± 0.12	0.35 ± 0.04
Low-fat milk | Beverage carton, flat top	0.35 ± 0.04	0.52 ± 0.16	0.43 ± 0.14
Low-fat milk | Beverage carton, gable top	0.40 ± 0.10	0.51 ± 0.09	0.45 ± 0.09
Whole milk | Beverage carton, gable top	0.28 ± 0.11	0.34 ± 0.07	0.31 ± 0.06
Whole milk | Glass bottle	0.31 ± 0.05	0.33 ± 0.05	0.32 ± 0.03

**Note:**

Results are given in percent, variability of results in percentage points.

**Figure 8:**
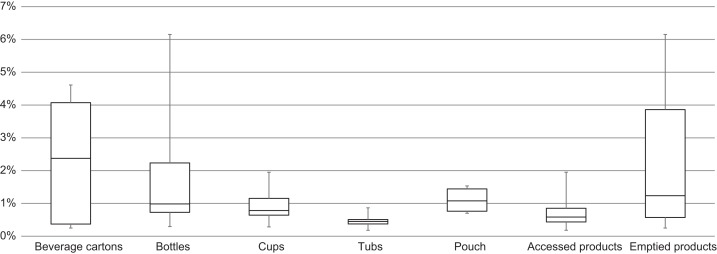
Emptiability results, grouped by types of packaging.

**Figure 9:**
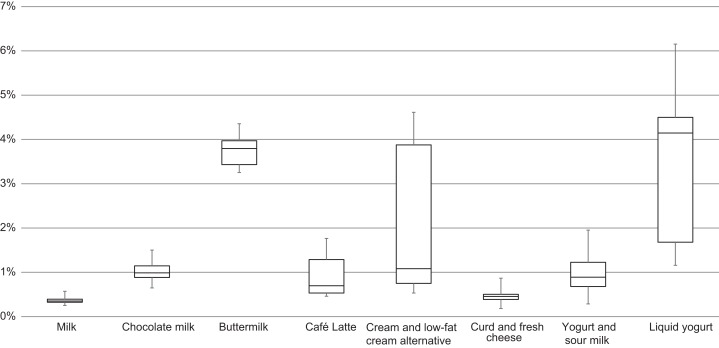
Emptiability results, grouped by types of dairy products.

From comparing different food products and types of packaging it is obvious that the range of technical emptiability of the same packaging with different filling goods (Fig. 8), as well as of the same food in different packaging (Fig. 9) has a wide range of margin. This indicates that technical emptiability is a result of a food-packaging combination rather than of food or packaging properties solely. However, from Fig. 8 it is apparent that technical emptiability of dairy products in packaging that was emptied is better than in packaging that was accessed (i.e., spooned out).

In the case of low-fat cream alternative, the exact same food product is available in two different types of packaging (bottle and pouch). Here, the emptiability of the pouch is distinctly better due to its ability to be squeezed after pouring out the contents. Furthermore, buttermilk in beverage cartons with gable tops seems to have slightly better emptiability than in those with bottle-shaped tops.

For the most part, there are differences in emptiability results for 22 and 7 °C, particularly for café latte in a cup and low-fat cream alternative in a pouch. Nevertheless, no clear positive or negative trend between temperature and emptiability can be observed (Mann–Whitney-U test: *U* = 641; *p* = 0.94).

### Streamlined LCA results

Food comprises the largest percentage of each package examined, whereas primary packaging generally has a small contribution to the overall results, ranging from 1.6% to 52.4% for climate change (Data S3) (mean 12.8%, median 9.9%). The obtained streamlined LCA results of primary packaging are in line with other studies that report average climate change values of 5.0% (Silvenius et al., 2011), or between 7.0% and 13.9% in the case of milk packaging (Licciardello, 2017).

For all six impact categories identified as relevant, the impacts of primary packaging increase after including technical emptiability (Tables 3 and 4). These range from +1% (±0) for the fossil use of fresh cheese to +1,827% (±141) for the terrestrial eutrophication of cream in a beverage carton.

**Table 3:** Percentage increase in streamlined LCA results of primary packaging for milk and milk-based drinks due to technical emptiability.

Dairy product	Acidification	Respiratory effects, inorganics	Climate change	Eutrophication, terrestrial	Eutrophication, freshwater	Resource use, fossils
Cafe Latté | PET bottle	22 ± 3	18 ± 3	3 ± 0	50 ± 7	3 ± 0	1 ± 0
Cafe Latté | PP cup	78 ± 34	45 ± 19	10 ± 4	164 ± 72	13 ± 6	4 ± 2
Chocolate milk | Beverage carton, flat top	103 ± 21	44 ± 9	28 ± 6	181 ± 37	24 ± 5	8 ± 2
Chocolate milk | PET bottle, variant a	41 ± 7	37 ± 7	6 ± 1	89 ± 16	5 ± 1	2 ± 0
Chocolate milk | PET bottle, variant b	45 ± 6	37 ± 5	7 ± 1	100 ± 13	6 ± 1	3 ± 0
L-free skimmed milk | Beverage carton, gable top	43 ± 5	18 ± 2	9 ± 1	77 ± 9	9 ± 1	3 ± 0
Low-fat milk | Beverage carton, flat top	38 ± 13	18 ± 6	8 ± 2	74 ± 24	7 ± 2	3 ± 1
Low-fat milk | Beverage carton, gable top	33 ± 7	15 ± 3	7 ± 1	63 ± 13	6 ± 1	2 ± 1
Whole milk | Beverage carton, gable top	46 ± 9	20 ± 4	9 ± 2	83 ± 16	10 ± 2	3 ± 1
Whole milk | Glass bottle	5 ± 0	3 ± 0	1 ± 0	11 ± 1	1 ± 0	1 ± 0

**Note:**

Results are given in percent, variability of results in percentage points.

**Table 4:** Percentage increase in streamlined LCA results of primary packaging for dairy products other than milk due to technical emptiability.

Dairy product	Acidification	Respiratory effects	Climate change	Eutrophication, terrestrial	Eutrophication, freshwater	Resource use, fossils
Buttermilk | Beverage carton, bottle top, variant a	279 ± 31	125 ± 14	51 ± 6	512 ± 56	66 ± 7	19 ± 2
Buttermilk | Beverage carton, bottle top, variant b	272 ± 27	121 ± 12	50 ± 5	498 ± 50	65 ± 6	19 ± 2
Buttermilk | Beverage carton, gable top	243 ± 9	106 ± 4	49 ± 2	437 ± 16	55 ± 2	17 ± 1
Cream, 23% fat | Beverage carton, flat top	1,045 ± 81	426 ± 33	264 ± 20	1,827 ± 141	208 ± 16	72 ± 6
Cream, 36% fat | HDPE bottle	99 ± 8	68 ± 5	8 ± 1	245 ± 20	34 ± 3	3 ± 0
Cream, 36% fat | PS cup	165 ± 28	123 ± 21	15 ± 2	431 ± 73	60 ± 10	7 ± 1
Curd cheese, creamy | PS tub	65 ± 20	47 ± 15	8 ± 2	166 ± 52	25 ± 8	4 ± 1
Curd cheese, crumbly | PS tub	25 ± 11	18 ± 8	3 ± 1	63 ± 28	10 ± 4	2 ± 1
Fresh cheese, herbs | PP tub	62 ± 22	44 ± 15	6 ± 2	152 ± 53	19 ± 7	3 ± 1
Fresh cheese, radish | PP tub, variant a	41 ± 11	30 ± 8	5 ± 1	95 ± 25	10 ± 3	2 ± 1
Fresh cheese, radish | PP tub, variant b	44 ± 7	32 ± 5	5 ± 1	104 ± 16	17 ± 3	3 ± 0
Fresh cheese, sweet pepper | PP tub	44 ± 9	31 ± 7	5 ± 1	108 ± 23	14 ± 3	2 ± 0
Fresh cheese, sweetened | PS cup	36 ± 7	28 ± 6	4 ± 1	93 ± 18	37 ± 7	2 ± 0
Liquid yogurt | Beverage carton, bottle top	390 ± 19	170 ± 8	87 ± 4	700 ± 33	121 ± 6	35 ± 2
Liquid yogurt | Beverage carton, gable top	318 ± 28	134 ± 12	87 ± 8	555 ± 50	99 ± 9	34 ± 3
Liquid yogurt, blueberries | PET bottle	86 ± 7	73 ± 6	18 ± 1	184 ± 15	21 ± 2	13 ± 1
Liquid yogurt, strawberry | PET bottle	37 ± 5	29 ± 4	6 ± 1	82 ± 12	8 ± 1	4 ± 1
Liquid yogurt, vanilla | PET bottle	76 ± 19	61 ± 15	12 ± 3	172 ± 42	14 ± 3	6 ± 1
Low-fat cream alternative | PET bottle	29 ± 1	25 ± 1	7 ± 0	61 ± 1	7 ± 0	5 ± 0
Low-fat cream alternative | Polymer pouch	62 ± 31	51 ± 25	6 ± 33	146 ± 73	24 ± 12	3 ± 2
Sour milk | PP cup	28 ± 20	14 ± 10	5 ± 1	57 ± 40	7 ± 5	3 ± 2
Sour milk | PS cup	36 ± 7	27 ± 5	4 ± 1	91 ± 17	22 ± 4	3 ± 1
Yogurt, cereals | PS cup	39 ± 3	31 ± 2	4 ± 0	103 ± 7	52 ± 4	2 ± 0
Yogurt, chocolate | PS cup	36 ± 10	26 ± 8	5 ± 2	88 ± 26	12 ± 3	3 ± 1
Yogurt, fruits | Twin-PP cup	49 ± 9	36 ± 6	7 ± 1	111 ± 20	11 ± 2	4 ± 1
Yogurt, vanilla | PS cup	63 ± 15	47 ± 11	8 ± 2	162 ± 39	32 ± 8	5 ± 1

**Note:**

Results are given in percent, variability of results in percentage points.

For climate change, the highest increase can be found for cream 23% fat (264% ± 20), liquid yogurt (87% ± 8) and buttermilk (51% ± 6) in beverage cartons. Regarding the impact on climate change, the inclusion of emptiability is of less relative importance for curd cheese, fresh cheese, and yogurt (3% ± 1 to 8% ± 2). This also applies to milk, particularly for whole milk in a glass bottle (1% ± 0), as well as to low-fat and skimmed milk in beverage cartons (7% ± 1 to 9% ± 1). For milk and milk-based drinks, emptiability is of higher importance for café latte in a PP cup (10% ± 4) and particularly for chocolate milk in a beverage carton (28% ± 6).

Acidification was identified as the most relevant impact category for the defined functional unit. In this category, beverage cartons with buttermilk, liquid yogurt and cream (1,045% ± 81) are again the packaging for which technical emptiability leads to the highest relative increase in impacts.

Different from the six relevant impact categories, also reductions of environmental impacts due to emptiability were calculated. This concerns the categories ozone layer depletion, land use and human toxicity (non-carcinogenic effects) (Data S3). The (imputed) decreases in ozone layer depletion and land use result mainly from the credits awarded by the incineration of packaging and thus the substitution of fossil fuels or biomass. The decrease in human toxicity is due to the heavy metal uptake of crops cultivated for animal feed contributing more to the overall impact than the generation of emissions or waste. Apart from this, too much emphasis should not be placed on toxicity indicators since they are currently not very robust and therefore excluded from being communicated or added to the PEF single score (Sala, Cerutti & Pant, 2018).

## Discussion

### Implications of technical emptiability

For the evaluation of the relevance of technical emptiability, existing LCA guidance documents may be used. ISO 14044 allows for a cut-off of inputs in LCA that contribute less than 1% to the total system regarding mass or energy (ISO 14044, 2006) while the PEF does not recommend any kind of cut-off in advance (European Commission, 2017). Additionally, the PEFCR for dairy products are encouraging the inclusion of primary data on FLW in LCA whenever available (Bengoa, Dubois & Humbert, 2018). According to ISO 14044 and its mass-related cut-off, food residues in the analyzed packages should be included except for milk, cream, cheese, sour milk, yogurt with cereals, and café latte in a PET bottle. However, following this approach would mean that the GWP^100^ of, for example, a beverage carton for cream would be understated by the factor 2.6. Defining relevance as a percentage increase of under 5% after including food residues, then only the technical emptiability of four out of 36 analyzed products could be seen as insignificant for climate change results of primary packaging. However, after including more impact categories besides climate change, technical emptiability is relevant for every analyzed packaging and thus should definitely be considered in future studies.

The results show clearly that food residues are particularly important for resource-intensive foods and for packaging, which is resource-friendly, such as the beverage carton. Thus, for whole milk in a glass bottle, the relative contribution of food residues to the overall environmental impact of packaging is of less importance.

Additionally, it should be stated that while the increase of environmental impacts is certainly relevant when looking at packaging alone, the picture is different for the whole life cycle of the products, that is, after the food production is included. Here, the difference in environmental impacts between “one kg distributed food” and “one kg consumed food” only ranges from 0% to +2% for all products (Data S3). This shows once again that the production of food leads to much greater environmental impacts than that of packaging.

### Limitations regarding emptiability testing

In this study, a method to operationalize technical emptiability was proposed, a packaging attribute that clearly distinguishes itself from the potentially wasteful behavior of a consumer. Yet, while the results for products which are to be emptied (e.g., bottles) do not depend on the meticulousness of the person performing the tests, this may be different for spooned out products.

The temperature at which dairy products are consumed could be either room temperature, refrigerator temperature or somewhere in between. Therefore, the mean of both EMPT_22 °C_ and EMPT_7 °C_ was used, thus disregarding differences between temperatures. In future studies of products that are consumed only at a specific temperature, emptiability testing should also focus on this temperature.

The aim of this explorative study was to cover a broad range of products. With *n* = 3, this already resulted in 216 tests for the 36 products analyzed at both temperatures. As a result, the variability of technical emptiability for some products was quite large.

In future emptiability studies, the sample size should be increased if results with lower variability are required.

### Limitations regarding the evaluation of environmental impacts

No primary data was used for the calculations of the streamlined LCA of packaging and dairy products. Therefore, the actual environmental impacts of the investigated products may actually be lower or higher, depending on the energy and resource efficiency of the respective companies. Furthermore, the exact composition of dairy products was not known. Particularly, the amount of milk required for the production of these was estimated by dry mass allocation. Due to the fact that milk is the most environmentally substantial input in dairy products (Famiglietti et al., 2019), an over- or underestimation could have a major impact on the results.

For the assessment of EVOH used in the multi-layer polymer pouch, the dataset of ethylene vinyl acetate (EVA) was used as a proxy, hence not considering an otherwise additionally necessary production process. However, EVOH accounts for only 3% of its mass. Furthermore, a previous LCA study reports using EVA as a proxy as acceptable (Humbert et al., 2009).

Another limitation of this study is that only a generic dataset for the incineration of food was used, thus neglecting differences of food properties (i.e., lower heating values). Still, this life cycle stage only contributes a maximum of 0.24% to the overall climate change results and even less to other impact categories.

## Conclusions

The present results show that in the food-packaging system of dairy products in Austria, the food contents always account for the highest percentage in LCA results. Surprising is that for some products, food residues are responsible for even higher environmental impacts than their primary packaging. It should be noted that the goal of this study was to represent the “ideal consumer.” Thus, the results for “practical emptiability” are probably even higher than for the derived technical emptiability, since it can be assumed that consumers are not emptying packages as meticulously as did the authors of this paper. Future research should be concerned with not only extending the method on testing technical emptiability in this paper, but also on finding approaches on how to measure practical emptiability.

Furthermore, it was not the focus of the study to investigate the relationship between packaging design and technical emptiability, which should also be addressed in future research. Nevertheless, the results of this study indicate that food products are at greater risk of causing higher amounts of food residues if there are contained in packaging where the contents are not easy to access (i.e., bottles and beverage cartons).

Presumably, food residues in packaging cause severe economic and ecological implications as food lost due to poor emptiability may add up to vast quantities for whole markets. Furthermore, European countries are obliged to introduce plastic packaging which is 100% recyclable to the market and to drastically increase the recycling rate of plastic packaging by 2030 (European Commission, 2018; European Parliament, 2018). Hence, packaging designers should develop packaging with good emptiability in addition to good recyclability due to the interference of food residues with the recycling process (Meurer et al., 2017; Maris et al., 2018). Measuring technical emptiability is only possible with already existing packaging, hence making a priori evaluation difficult. However, some packaging features can already be considered by designers, such as the use of wide necks or designing packaging that can be stood upside down (Packaging SA, 2017).

Several LCA analysts (Flysjö, 2011; Williams & Wikström, 2011; Grant, Barichello & Fitzpatrick, 2015; Manfredi et al., 2015; Verghese et al., 2015; Gruber et al., 2016; Heller, Selke & Keoleian, 2018) already incorporate FLW into their work on packaging. Food residues can have substantial environmental impacts, and in some cases even greater than that of the respective packaging. Thus, it is crucial that future comparative studies of packaging also include emptiability, since this could change the identification of the most environmentally friendly option.

## Supplemental Information

10.7717/peerj.7578/supp-1 Supplemental Information 1 Details of emptiability results.

10.7717/peerj.7578/supp-2 Supplemental Information 2 Descriptions of dairy products, packaging and LCA datasets. Type and mass of each packaging material, dairy product ingredients and Ecoinvent datasets used for each product

10.7717/peerj.7578/supp-3 Supplemental Information 3 Streamlined LCA results. Results for each impact category and life cycle stage

10.7717/peerj.7578/supp-4 Supplemental Information 4 Life cycle inventory of Austrian fat-protein corrected milk (FPCM).

## Additional Information and Declarations

### Data Availability

The following information was supplied regarding data availability: Tables with descriptions of all dairy products regarding food (nutritional values, list of ingredients) and packaging (material and mass of individual packaging components) are available in the Supplemental Files.
