# Supplementary material for: Technical emptiability of dairy product packaging and its environmental implications in Austria
Source: PeerJ. 2019 Sep 10;7:e7578. doi: 10.7717/peerj.7578 (PMC6743449; doi:10.7717/peerj.7578)
Supplement: Supplemental Information 4 [file peerj-07-7578-s004.docx]

| **Inputs** | **Unit** | **Amount** | **Outputs** | **Unit** | **Amount** |
| --- | --- | --- | --- | --- | --- |
| alfalfa-grass silage | kg | 0.0183312 | Ammonia | kg | 0.01304 |
| barley grain, feed | kg | 0.0098707 | Dinitrogen monoxide | kg | 0.000810282 |
| grass silage, organic | kg | 0.0282019 | Methane, biogenic | kg | 0.01645064 |
| grass, Swiss integrated production | kg | 0.2115142 | NMVOC, non-methane volatile organic compounds, unspecified origin | kg | 0.000102919 |
| hay | kg | 0.2072839 | Particulates, < 10 um | kg | 7.52259E-10 |
| limestone, crushed, washed | kg | 0.00164 | Particulates, < 2.5 um | kg | 1.67169E-10 |
| magnesium oxide | kg | 0.000270372 | Particulates, > 10 um | kg | 7.52259E-10 |
| maize grain, feed | kg | 0.0282019 | Fat and protein corrected milk, from cow | kg | 1.00 |
| maize silage | kg | 0.0705047 |  |  |  |
| maize starch | kg | 0.0169211 |  |  |  |
| operation, housing system, cattle,  loose, per animal unit | Item(s) | 1.17411E-05 |  |  |  |
| operation, housing system, cattle,  tied, per animal unit | Item(s) | 0.000116158 |  |  |  |
| rape meal | kg | 0.0183312 |  |  |  |
| selenium | kg | 1.62223E-07 |  |  |  |
| sodium chloride, powder | kg | 0.001638618 |  |  |  |
| soybean meal | kg | 0.0183312 |  |  |  |
| straw | kg | 0.0141009 |  |  |  |
| transport, passenger car, large size,  petrol, EURO 4 | km | 0.0178247 |  |  |  |
| vinasse, from fermentation of sugar beet | kg | 0.0070505 |  |  |  |
| wheat grain, feed | kg | 0.0564038 |  |  |  |
| wood chips, dry, measured as dry mass | kg | 0.046320583 |  |  |  |
